# Supplementary material for: Patterns and drivers of genome-wide codon usage bias in the fungal order Sordariales
Source: DNA Res. 2025 Dec 2;32(6):dsaf036. doi: 10.1093/dnares/dsaf036 (PMC12730877; doi:10.1093/dnares/dsaf036)
Supplement: dsaf036_Supplementary_Data [file dsaf036_supplementary_data.zip › supplementary figures.pdf]

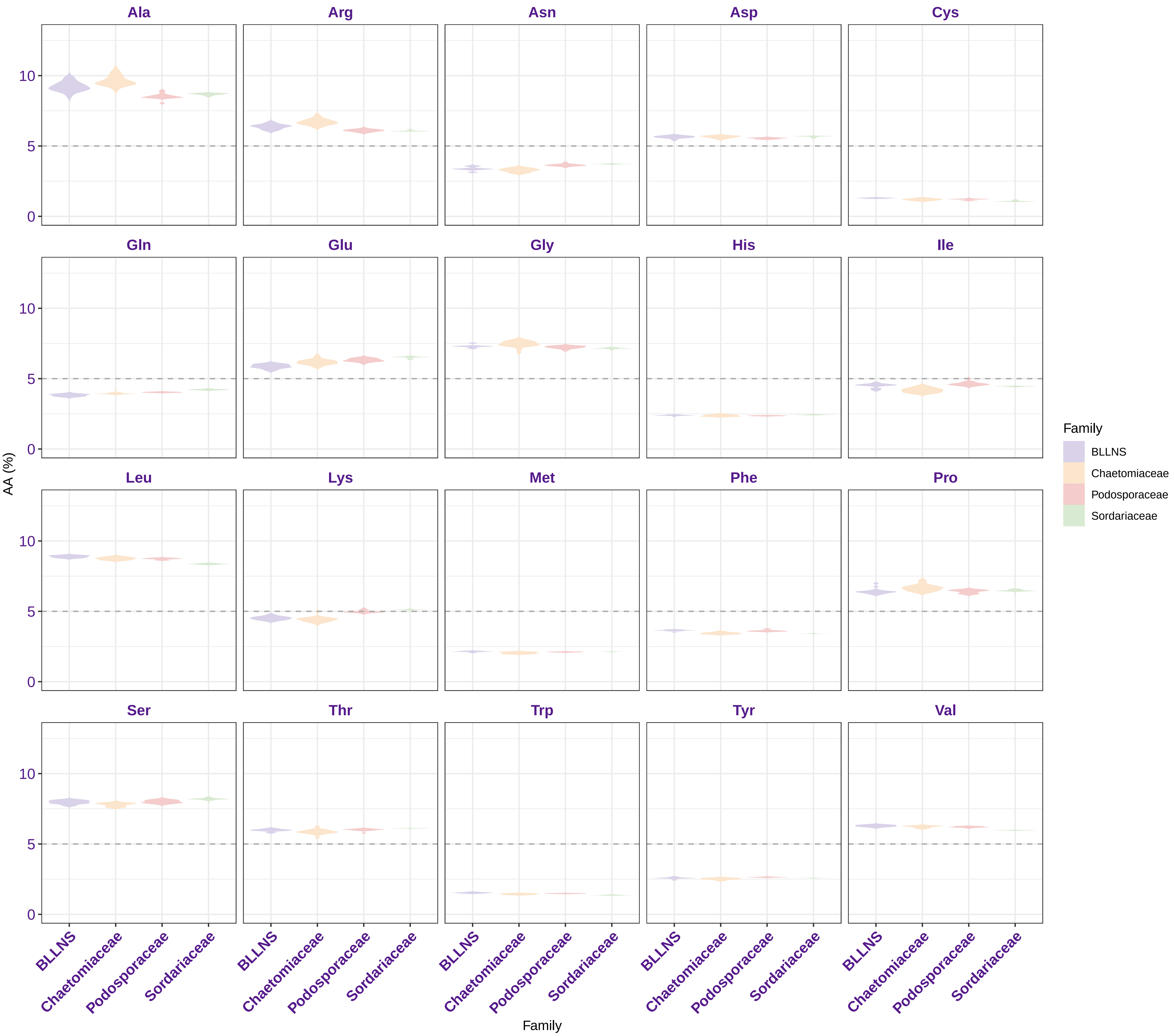

Ala
Arg
Asn
Asp
Cys
Gln
Glu
Gly
His
Ile
Leu
Lys
Met
Phe
Pro
Ser
Thr
Trp
Tyr
Val
AA (%)
0
5
10
BLLNS
Chaetomiaceae
Podosporaceae
Sordariaceae
Family

Figure S1: patterns of amino acid levels across Sordariales.
Violin plots for amino acid levels Chaetomiaceae, Podosporaceae, Sordariaceae, and the BLLNS. Significant difference was found between all groups for every amino acid (Kruskal Wallis p-value < 0.05 for each amino acid), and for the majority of 1:1 comparisons (Wilcoxon rank sum test with bonferonni p-value adjustment method, table S5). Grey dashed line indicates 5%, the percentage expected if each amino acid would be used equally.

Codon Usage (RSCU) by Family − Amino acids Ala, Leu, Ser, Gly, Pro

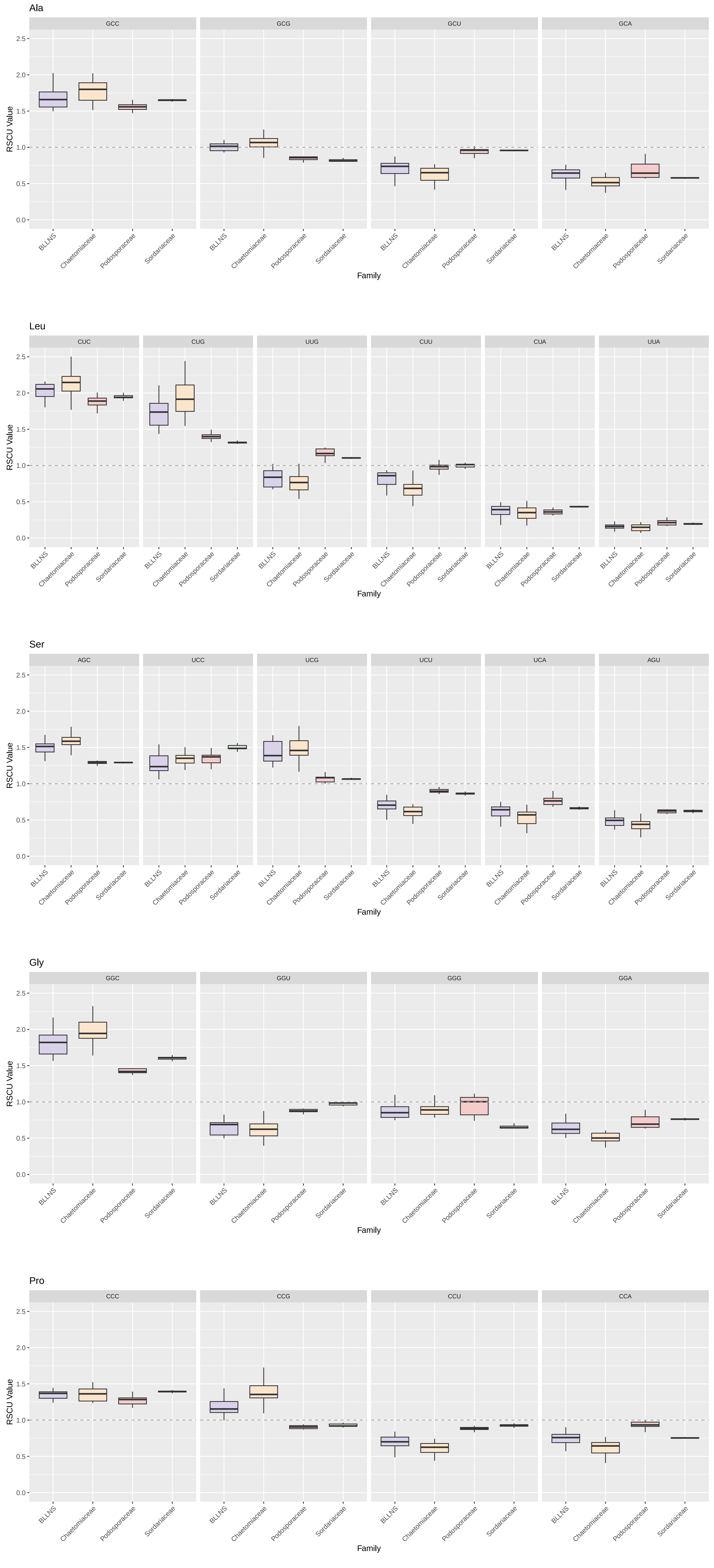

Codon Usage (RSCU) by Family − Amino acids Glu, Arg, Val, Thr, Asp

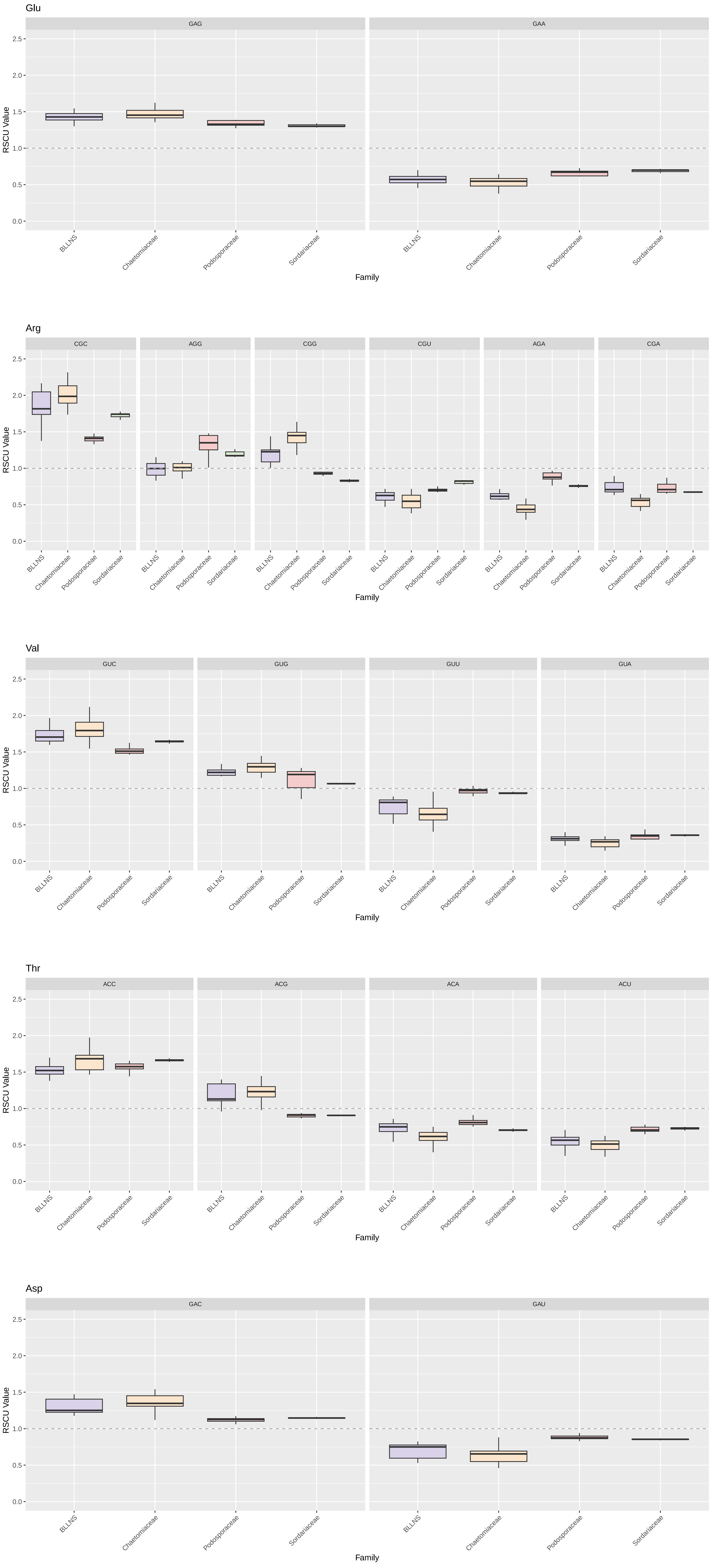

Codon Usage (RSCU) by Family − Amino acids Lys, Ile, Gln, Asn, Phe

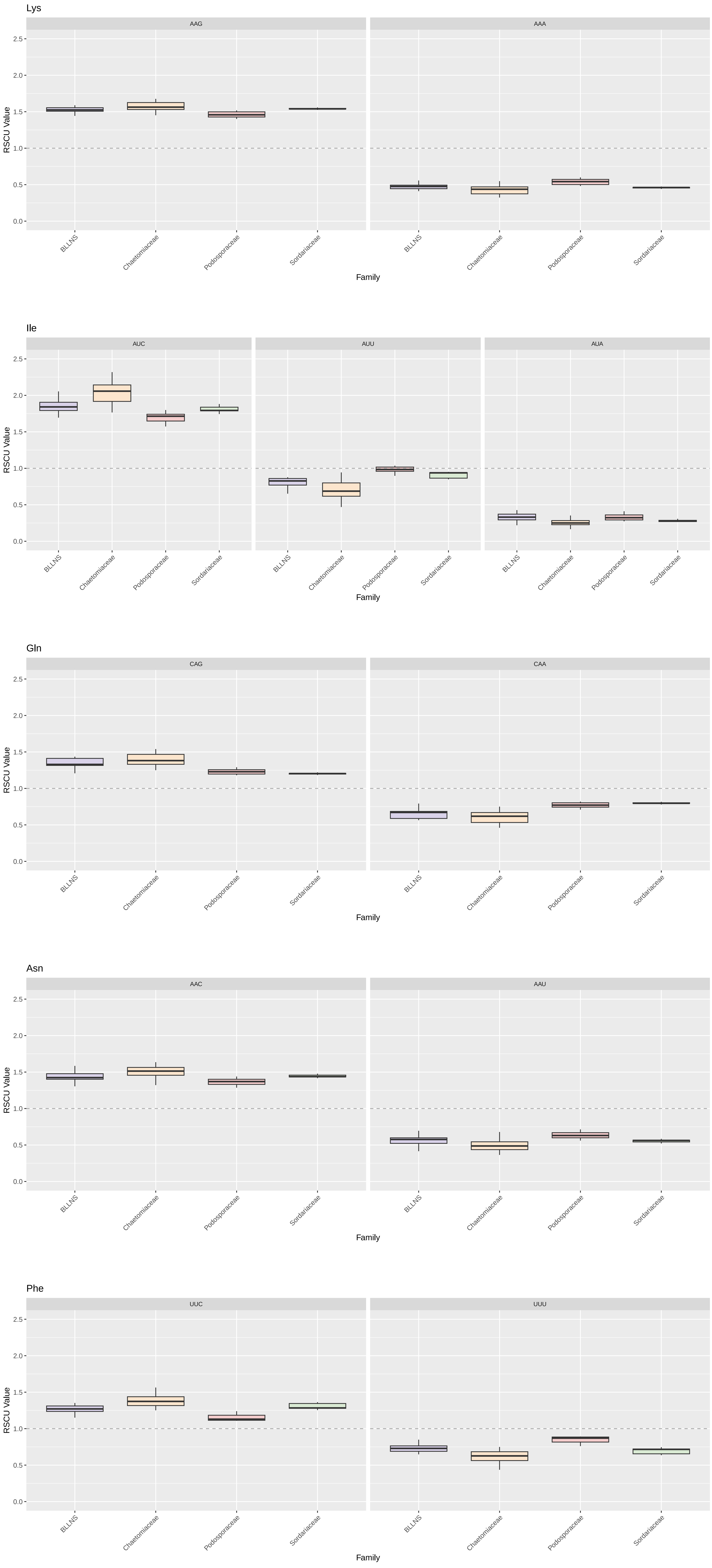

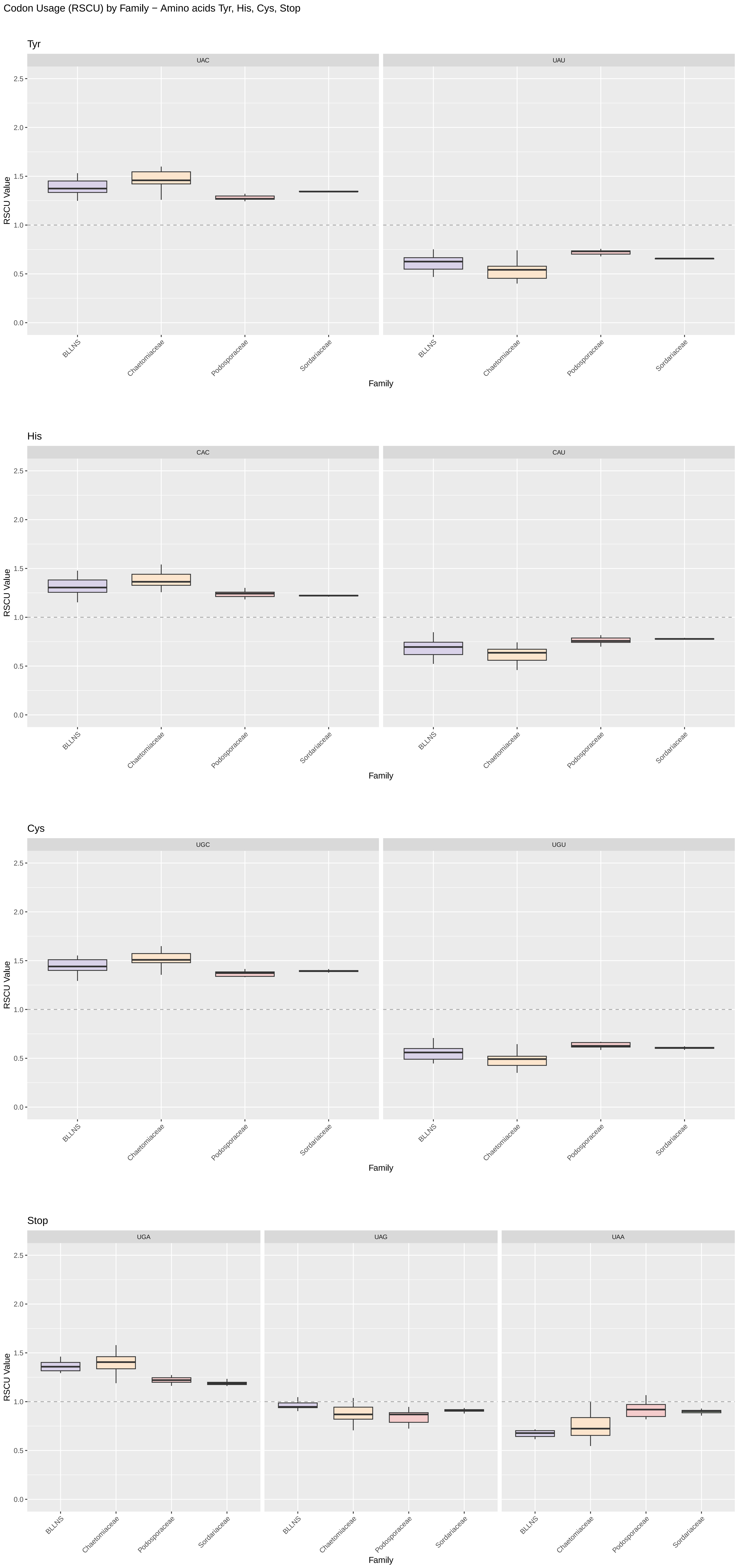

Codon Usage (RSCU) by Family − Amino acids Tyr, His, Cys, Stop
Tyr
UAC
UAU
His
CAC
CAU
Cys
UGC
UGU
Stop
UGA
UAG
UAA
RSCU Value
Family
BLLNS
Chaetomiaceae
Podosporaceae
Sordariaceae
0.0
0.5
1.0
1.5
2.0
2.5

Figure S2: patterns of codon usage across Sordariales.

Boxplots plots for RSCU values of codons for Chaetomiaceae, Podosporaceae, Sordariaceae, and the BLLNS. Significant difference was found between all groups for every codon (Kruskal Wallis p-value < 0.05 for each codon), and for the majority of 1:1 comparisons (Wilcoxon rank sum test with bonferonni p-value adjustment method, table S7). Grey dashed line indicates 1, the RSCU value expected if codon would be used equally to encode for a certain amino acid.

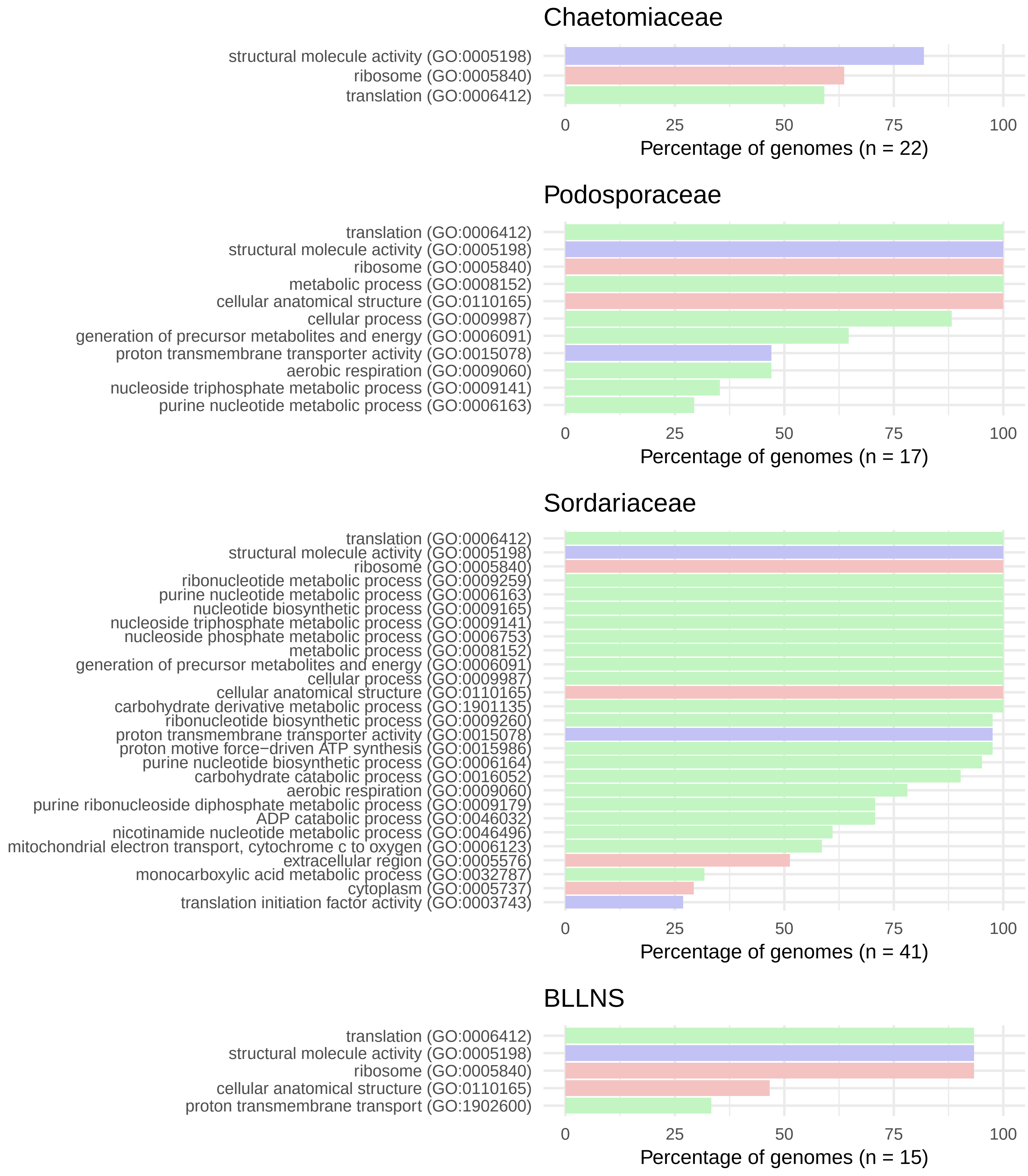

Chaetomiaceae
structural molecule activity (GO:0005198)
ribosome (GO:0005840)
translation (GO:0006412)
0
25
50
75
100
Percentage of genomes (n = 22)
Podosporaceae
translation (GO:0006412)
structural molecule activity (GO:0005198)
ribosome (GO:0005840)
metabolic process (GO:0008152)
cellular anatomical structure (GO:0110165)
cellular process (GO:0009987)
generation of precursor metabolites and energy (GO:0006091)
proton transmembrane transporter activity (GO:0015078)
aerobic respiration (GO:0009060)
nucleoside triphosphate metabolic process (GO:0009141)
purine nucleotide metabolic process (GO:0006163)
0
25
50
75
100
Percentage of genomes (n = 17)
Sordariaceae
translation (GO:0006412)
structural molecule activity (GO:0005198)
ribosome (GO:0005840)
ribonucleotide metabolic process (GO:0009259)
purine nucleotide metabolic process (GO:0006163)
nucleotide biosynthetic process (GO:0009165)
nucleoside triphosphate metabolic process (GO:0009141)
nucleoside phosphate metabolic process (GO:0006753)
metabolic process (GO:0008152)
generation of precursor metabolites and energy (GO:0006091)
cellular process (GO:0009987)
cellular anatomical structure (GO:0110165)
carbohydrate derivative metabolic process (GO:1901135)
ribonucleotide biosynthetic process (GO:0009260)
proton transmembrane transporter activity (GO:0015078)
proton motive force−driven ATP synthesis (GO:0015986)
purine nucleotide biosynthetic process (GO:0006164)
carbohydrate catabolic process (GO:0016052)
aerobic respiration (GO:0009060)
purine ribonucleoside diphosphate metabolic process (GO:0009179)
ADP catabolic process (GO:0046032)
nicotinamide nucleotide metabolic process (GO:0046496)
mitochondrial electron transport, cytochrome c to oxygen (GO:0006123)
extracellular region (GO:0005576)
monocarboxylic acid metabolic process (GO:0032787)
cytoplasm (GO:0005737)
translation initiation factor activity (GO:0003743)
0
25
50
75
100
Percentage of genomes (n = 41)
BLLNS
translation (GO:0006412)
structural molecule activity (GO:0005198)
ribosome (GO:0005840)
cellular anatomical structure (GO:0110165)
proton transmembrane transport (GO:1902600)
0
25
50
75
100
Percentage of genomes (n = 15)

Figure S3: GO term enrichment analysis of genes under selection for codon usage, summarized per family. Percentages are based on how many genomes each term was found to be significantly enriched in. GO terms are colored based on their top-level categories (green: Biological Process, red: Cellular Component, blue: Molecular Function). For visualization, only GO terms enriched in at least 25% of the genomes of each family are shown.

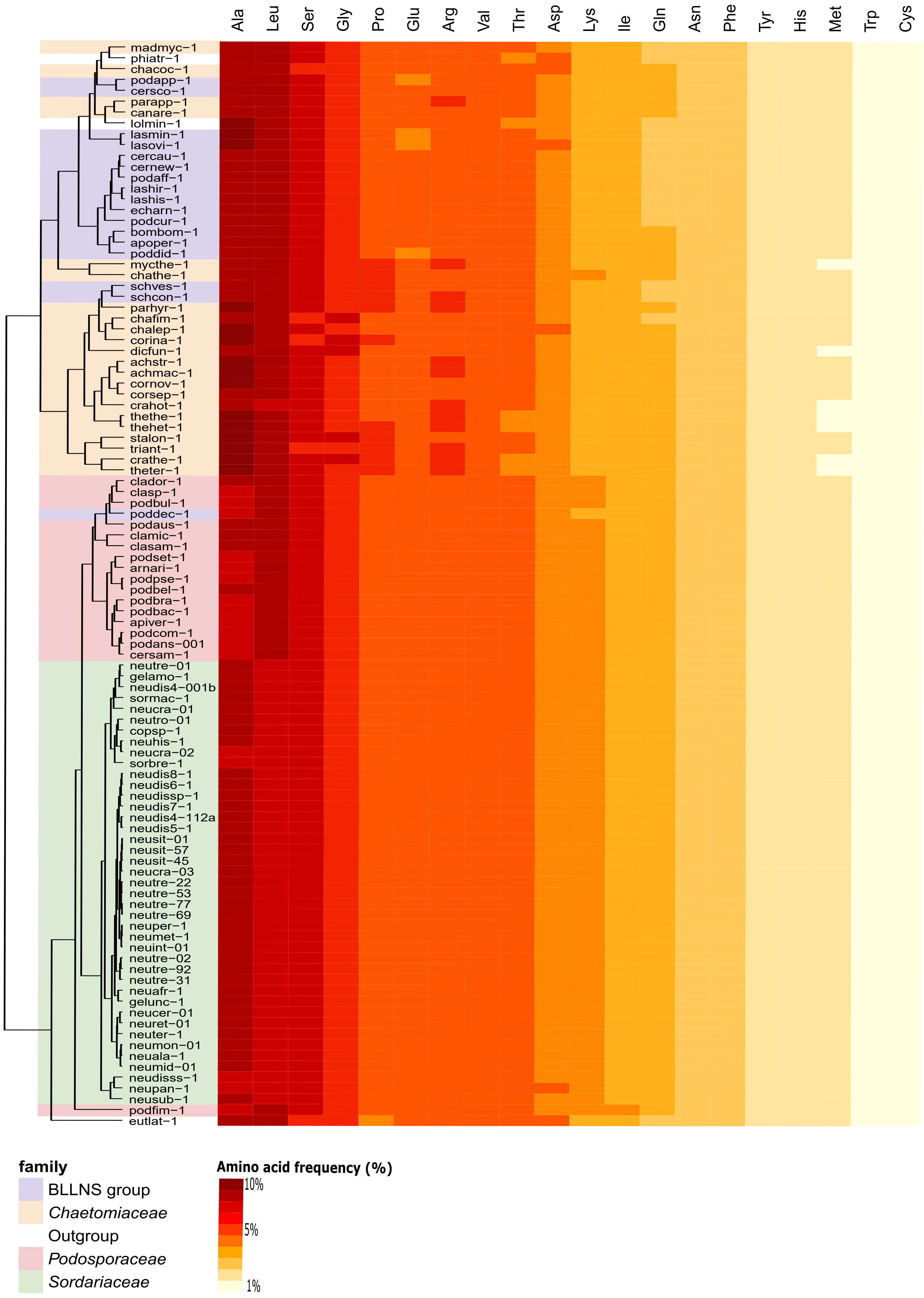
Ala
Leu
Ser
Gly
Pro
Glu
Arg
Val
Thr
Asp
Lys
Ile
Gln
Asn
Phe
Tyr
His
Met
Trp
Cys
madmyc−1
phiatr−1
chacoc−1
podapp−1
cersco−1
parapp−1
canare−1
lolmin−1
lasmin−1
lasovi−1
cercau−1
cernew−1
podaff−1
lashir−1
lashis−1
echarn−1
podcur−1
bombom−1
apoper−1
poddid−1
mycthe−1
chathe−1
schves−1
schcon−1
parhyr−1
chafim−1
chalep−1
corina−1
dicfun−1
achstr−1
achmac−1
cornov−1
corsep−1
crahot−1
thethe−1
thehet−1
stalon−1
triant−1
crathe−1
theter−1
clador−1
clasp−1
podbul−1
poddec−1
podaus−1
clamic−1
clasam−1
podset−1
arnari−1
podpse−1
podbel−1
podbra−1
podbac−1
apiver−1
podcom−1
podans−001
cersam−1
neutre−01
gelamo−1
neudis4−001b
sormac−1
neucra−01
neutro−01
copsp−1
neuhis−1
neucra−02
sorbre−1
neudis8−1
neudis6−1
neudissp−1
neudis7−1
neudis4−112a
neudis5−1
neusit−01
neusit−57
neusit−45
neucra−03
neutre−22
neutre−53
neutre−77
neutre−69
neuper−1
neumet−1
neuint−01
neutre−02
neutre−92
neutre−31
neuafr−1
gelunc−1
neucer−01
neuret−01
neuter−1
neumon−01
neuala−1
neumid−01
neudisss−1
neupan−1
neusub−1
podfim−1
eutlat−1
family
BLLNS group
Chaetomiaceae
Outgroup
Podosporaceae
Sordariaceae
Amino acid frequency (%)
10%
5%
1%

Figure S4: Amino acid composition across the Sordariales, with automatic eucladian clustering of genomes. Average amino acid frequencies (%) for all genomes. Amino acids are ordered from most used (left, dark-red) to least used (right, light-yellow). Sordariaceae and Podosporaceae cluster together, and Chaetomiaceae and the BLLNS group cluster together. Species names are represented by alphanumeric IDs, which correspond to species names outlined in table S1.

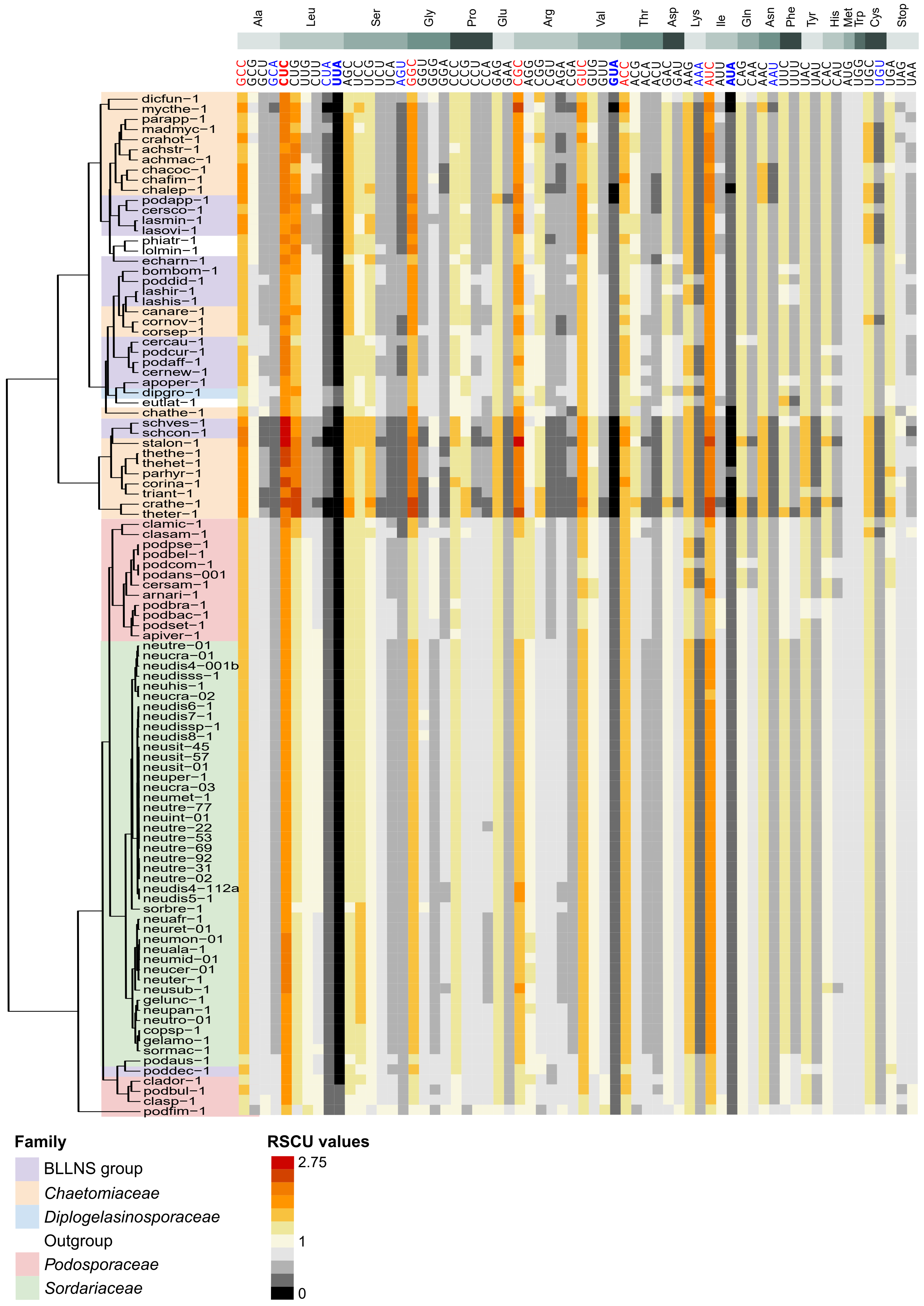
Ala
Leu
Ser
Gly
Pro
Glu
Arg
Val
Thr
Asp
Lys
Ile
Gln
Asn
Phe
Tyr
His
Met
Trp
Cys
Stop
GCC GCG GCU GCA CUC CUG UUG UUU CUU CUA UUA AGC UCC UCG UCU UCA AGU GGC GGU GGG GGA CCC CCG CCU CCA GAG GAA CGC AGG CGG CGU AGA CGA GUC GUG GUU GUA ACC ACG ACA ACU GAC GAU AAG AAA AUC AUU AUA CAG CAA AAC AAU UUC UUU UAC UAU CAC CAU AUG UGG UGC UGU UGA UAG UAA
dicfun−1
mycthe−1
parapp−1
madmyc−1
crahot−1
achstr−1
achmac−1
chacoc−1
chafim−1
chalep−1
podapp−1
cersco−1
lasmin−1
lasovi−1
phiatr−1
lolmin−1
echarn−1
bombom−1
poddid−1
lashir−1
lashis−1
canare−1
cornov−1
corsep−1
cercau−1
podcur−1
podaff−1
cernew−1
apoper−1
dipgro−1
eutlat−1
chathe−1
schves−1
schcon−1
stalon−1
thethe−1
thehet−1
parhyr−1
corina−1
triant−1
crathe−1
theter−1
clamic−1
clasam−1
podpse−1
podbel−1
podcom−1
podans−001
cersam−1
arnari−1
podbra−1
podbac−1
podset−1
apiver−1
neutre−01
neucra−01
neudis4−001b
neudisss−1
neuhis−1
neucra−02
neudis6−1
neudis7−1
neudissp−1
neudis8−1
neusit−45
neusit−57
neusit−01
neuper−1
neucra−03
neumet−1
neutre−77
neuint−01
neutre−22
neutre−53
neutre−69
neutre−92
neutre−31
neutre−02
neudis4−112a
neudis5−1
sorbre−1
neuafr−1
neuret−01
neumon−01
neuala−1
neumid−01
neucer−01
neuter−1
neusub−1
gelunc−1
neupan−1
neutro−01
copsp−1
gelamo−1
sormac−1
podaus−1
poddec−1
clador−1
podbul−1
clasp−1
podfim−1
Family
BLLNS group
Chaetomiaceae
Diplogelasinosporaceae
Outgroup
Podosporaceae
Sordariaceae
RSCU values
2.75
1
0

Figure S5: Patterns of relative synonymous codon usage, with automatic eucladian clustering of genomes. Amino acids are ordered in the same order as figure 1, with individual codons ordered as in figure 2. RSCU values larger than 1 indicate that there is a higher frequency of a particular codon in the genome than expected under random use, while RSCU values <1 indicate that a codon is less frequent within the genome. Codons with RSCU values >1.6 or <0.6 are seen as over- and underrepresented codons. Heatmap colors range from blackgrey (RSCU <1), to white (RSCU = 1), to red-darkred (RSCU>1). Species names are represented by alphanumeric IDs, which correspond to species names outlined in table S1.
